# Assessment of *CRB1*-Associated Retinopathies Using the S-MAIA Fast Protocol and Spectral-Domain Optical Coherence Tomography

**DOI:** 10.3390/biomedicines13030555

**Published:** 2025-02-21

**Authors:** Bethany E. Higgins, Ana Catalina Rodriguez-Martinez, Giovanni Montesano, Vijay K. Tailor-Hamblin, Samantha Malka, Robert H. Henderson, Mariya Moosajee

**Affiliations:** 1 UCL Institute of Ophthalmology, London EC1V 9EL, UK; bethany.higgins@ucl.ac.uk (B.E.H.); giovmontesano@gmail.com (G.M.); vijay.tailor@ucl.ac.uk (V.K.T.-H.); 2 Moorfields Eye Hospital NHS Foundation Trust, London EC1V 2PD, UK; samantha.malka@nhs.net (S.M.); robert.henderson@gosh.nhs.uk (R.H.H.); 3 Great Ormond Street Hospital for Children NHS Foundation Trust, London WC1N 3JH, UK; 4 UCL-GOSH Institute of Child Health, London WC1N 1EH, UK; 5 The Francis Crick Institute, London NW1 1AT, UK

**Keywords:** crumbs cell polarity complex component 1 gene (*CRB1*), Leber congenital amaurosis (LCA), cone-rod dystrophy (CORD), early-onset severe retinal dystrophy (EOSRD), macular dystrophy (MD), spectral domain optical coherence tomography (SD-OCT), macular integrity assessment (S-MAIA)

## Abstract

**Background:** A cross-sectional study was conducted at Moorfields Eye Hospital, UK, involving patients with *CRB1*-associated retinopathies: macular dystrophy (MD), cone-rod dystrophy (CORD), and early-onset severe retinal dystrophy/Leber congenital amaurosis (EOSRD/LCA). The study aimed to evaluate *CRB1*-associated retinopathies using microperimetry (macular integrity assessment (S-MAIA) fast protocol) and spectral domain optical coherence tomography (SD-OCT). **Methods:** Data quality and participant attrition were assessed in 18 patients (10 MD, 5 EOSRD/LCA, 3 CORD), aged 10–52 years, with a median best corrected visual acuity (BCVA) of 0.41 logMAR. **Results:** Microperimetry and SD-OCT data were obtained from 14 and 18 patients, respectively, but eccentric fixation hindered structure-function analysis. All participants showed overall abnormal sensitivity on the S-MAIA fast protocol. Parafoveal volume was significantly increased, while foveal thickness and volume were reduced compared to normative data (*p* < 0.01). **Conclusions:** This study highlights the challenges of participant attrition and the need for alternative functional metrics to complement traditional evaluations. It also reinforces previous findings of abnormal retinal architecture in *CRB1*-associated retinopathies, providing further insights into S-MAIA and SD-OCT assessments for this patient population.

## 1. Introduction

The crumbs cell polarity complex component 1 gene (*CRB1;* OMIM #604210) is crucial for both the development and integrity of the retina [1]. When its function is impaired, it can result in various phenotypes, including Leber congenital amaurosis (LCA), early-onset severe retinal dystrophy (EOSRD), retinitis pigmentosa (RP), cone-rod dystrophy (CORD), and macular dystrophy (MD) [2], manifesting across a wide age range. Distinctive signs of *CRB1*-associated retinopathies include nummular pigmentation, fine yellow punctate deposits, preserved para-arteriolar retinal pigment epithelium, coarse and abnormal retinal lamination and thickened retina seen on spectral domain optical coherence tomography (SD-OCT) [1,3].

In clinical trials aiming to address *CRB1*-associated retinopathies, the necessity for robust and reliable outcome measures is paramount. SD-OCT serves as a crucial structural imaging modality in evaluating retinal architecture. By providing high-resolution cross-sectional images of the retina, it allows for detailed assessment of retinal layers, thickness, and morphological changes associated with *CRB1-associated* retinopathies [4].

Alongside SD-OCT, microperimetry is emerging as a promising clinical tool for assessing functional changes in the retina. It is a functional outcome metric for clinical trials approved by the US Food and Drug Administration and is being used in gene therapy trials [5,6]. Microperimetry has been described as a sensitive progression marker for individuals with *CRB1* with the potential to be a trial end point [2]. The macular integrity assessment (S-MAIA) maps macular sensitivity by integrating fundus imaging and eye tracking to account for eye movements. Testing protocols such as the fast (screening) protocol available for the S-MAIA enable rapid assessment of macular function. This protocol lasts approximately 3 min and is a suitable method to quickly identify abnormal retinal sensitivity within a battery of functional tests. This faster test is particularly beneficial for paediatric testing, where limited capacity for prolonged protocols poses challenges. While microperimetry using the MAIA and S-MAIA have been used to assess macular function in different inherited retinal diseases (IRDs) using longer testing protocols [7,8,9], the fast (screening) protocol has not yet been evaluated in this specific patient population.

This study primarily aims to assess retinal sensitivity using the S-MAIA and retinal volume using SD-OCT in a cohort of prospective patients with *CRB1-associated* retinopathies who were able to undergo best corrected visual acuity (BCVA) assessment using the ETDRS chart and had a baseline visual acuity in the better seeing eye of equal or better than 1.00 logMAR. Estimates of measurement properties of the devices and participant compliance to complete the examinations were also assessed. Furthermore, this study includes methodology to estimate reliability metrics (the rate of false positive [FP] responses) [10] when using the fast (screening) protocol of the S-MAIA.

## 2. Methods

### 2.1. Participant Recruitment and Genetic Testing

Participants were recruited from Moorfields Eye Hospital, London, United Kingdom. The inclusion criteria were to have molecularly confirmed biallelic *CRB1*-associated retinopathies (with either pathogenic or likely pathogenic variants) and BCVA of the better seeing eye ≤ 1.00 logMAR. All study participants gave informed consent to participate and all procedures were conducted in adherence to the tenets of the Declaration of Helsinki. The study received ethical approval and participants gave written informed consent for genetic testing, through the Genetic Study of Inherited Eye Disease (REC reference 12/LO/0141). No incentives for participation were offered, but travel expenses were reimbursed.

The methodology of genetic testing and interpretation at Moorfields Eye Hospital utilised in this study has been described previously [11]. DNA samples extracted from peripheral blood were used for genetic testing following informed consent. Molecular testing was performed in the clinical and research setting, using a targeted retinal gene panel through the Rare & Inherited Disease Genomic Laboratory at Great Ormond Street Hospital and whole genome sequencing (WGS) as part of the UK Genomics England 100,000 Genomes Project. The results were reviewed by experts to confirm variant pathogenicity, prevalence in publicly available genome databases, the clinical phenotype, and mode of inheritance prior to molecular diagnosis [12].

### 2.2. Phenotype Classification

Phenotyping of patients has been described previously [11]. In brief, patients were categorised into different phenotypes by clinicians (authors ARM and MM) via evidence from clinical data, retinal imaging, and age of onset. The phenotypes included in this study were EOSRD/LCA, MD, and CORD.

### 2.3. Clinical Assessment

A comprehensive ophthalmic assessment was carried out with functional visual testing and ophthalmic imaging as part of a prospective study of *CRB1* patients. Ophthalmic history and demographic details were collected from Moorfields Eye Hospital electronic patient records and via self-report during baseline assessment. BCVA was measured using the ETDRS chart one for the right eye, and chart two for the left eye. BCVA was converted to logarithmic minimum angle of resolution (LogMAR) for statistical analysis. Inter-ocular difference was calculated from baseline BCVA data and confirmed not to be significantly different (*p* = 0.26). As a result, measurements from the right eye were used for analysis.

Fundus-controlled microperimetry (macular integrity assessment (S-MAIA) perimeter; CenterVue-iCare, Padova, Italy) was used for the assessment of macular sensitivity. The 10-2 test grid (37 loci) was used to map patients’ macular sensitivity profile and the fast (screening) exam was chosen for due to its speed and its likelihood to be used in clinic. This is a suprathreshold test, whereby the sensitivity of each tested loci is not calculated, but an intensity of pre-determined brightness (in this case, 27 dB and 25 dB) is used at each test location. Tests where fixation losses were >15% were repeated. For functional analysis, the overall index of luminance sensitivity was computed as ‘normal’ (27 dB), ‘suspect’ (25 dB) or ‘abnormal’ (<25 dB). Numbers of ‘normal’, ‘suspect’, and ‘abnormal’ loci were also calculated. Fixation stability was measured through the calculation of 95% BCEA, encompassing 95% of all fixation points observed during the examination [13].

To assess test reliability of S-MAIA data, the rate of FP responses was estimated using a method adapted from Montesano et al. (2021) based on wrong pressure events, whereby a response occurs ≥1500 ms after a stimulus presentation and prior to the next stimulus presentation [14]. This method was found to be a better descriptor of the test performance than the commonly used blind spot response [10]. The methodology used to calculate this for the S-MAIA fast (screening) exam can be found in the Appendix A.

### 2.4. Eccentric Fixation

The preferred retinal locus (PRL) is a focal point on the retina used by individuals with central vision loss to fixate their gaze [15]. It serves as a substitute for the impaired fovea, enabling tasks like reading and object recognition. Fixation eccentricity measures the distance between the fovea and the PRL, providing insights into how individuals adapt to vision loss. To calculate eccentric fixation, the S-MAIA data were registered to SD-OCT images using an in-house application. The application matches the fundus pictures from the Spectralis and the MAIA using an affine transformation, calculated using the R package RNiftyReg (version 2.8.1; R Foundation for Statistical Computing).

### 2.5. Imaging Assessment

Macular SD-OCT scans were performed using the Spectralis device (Heidelberg Engineering, Heidelberg, Germany) within a 28 mm^2^ area, encompassing the standard 1, 3, and 6 mm grid template from the ETDRS. Specifically, volume scans were performed with a horizontal orientation (at 0°), covering an area of 20° × 20°. These scans comprised 97 B-scans, with a provision to reduce to 49 B-scans if optimal image quality was not achievable. In instances where ART (automatic real-time) eye tracking performance was compromised, the standard operating protocol allowed for adjustments to 9 frames or, if necessary, further reduction to 1 frame. Additionally, single-line scans with a visual field of 20° were conducted both horizontally (at 0°). The number of frames for these single-line scans was set at 100, incorporating 1536 A-scans. The integrated automatic segmentation Spectralis software (HEYEX2) was utilised for segmentation, with manual corrections made by author ARM for any segmentation errors.

For the structural analysis, full retinal volume data were extracted from both the central portion (CRT1mm) and the 3 mm inner ring of the ETDRS grid (CRT1–3mm). For reference, the CRT1mm is the central 1 mm ring centred on fovea, the CRT1–3mm is the 1–3 mm ring surrounding CRT1mm, and the CRT3–6mm is the 3–6 mm ring surrounding CRT1–3mm. Thickness data were also extracted from the central portion and foveal thickness was defined as the average thickness in the central 1000 μm diameter from the internal limiting membrane to Bruch’s membrane in a 1 mm diameter circle centred on the fovea of the ETDRS layout, taken from the point where six radial scans intersected [16]. The outer ring was excluded from the analysis due to missing data. For qualitative analysis, lamination of the retina was categorised into three groups, as detailed in previous work [17,18]. Retinal organisation and lamination were graded as follows: group 1, normal; group 2, normal organisation with coarse lamination; and group 3, disorganisation with coarse lamination.

### 2.6. Data Quality Control

For this study, only data from participants who had successfully recorded BCVA were considered. This additional inclusion criterion makes these results representative of a study population that can adequately perform chart-based visual function assessment. First, we identified missing data due to examination procedural errors (phase 1), for example, faults with the examination set-up. Next, we identified missing data for participant issues (phase 2, e.g., resulting from abandoned examination). Finally, we identified data deemed unreliable (phase 3, e.g., due to too many fixation errors or imaging artifacts). These results will be useful for those planning future studies/trials wanting to estimate attrition rates of data from people with *CRB1*-retinopathies.

### 2.7. Statistical Analysis

Analyses were performed using R statistical software, version 4.0.0 (R Foundation for Statistical Computing), and the appropriate parametric (*t*-test) and non-parametric (Mann–Whitney U test) statistics were applied after testing for normality. Bonferroni correction was used to adjust *p*-values for multiple comparisons. Linear regression was calculated to assess the relationship between eccentric fixation and BCVA, BCEA 95%, and foveal thickness. Correlational analyses were computed using Spearman’s test. Microperimetry and OCT data from the right eye only were used for analyses. Normative foveal thickness measures from 19 participants were sourced from Grover et al. and normative inner ring OCT volume parameters were taken from 20 participants from Murthy et al. to characterise these data [19,20]. While the age ranges of the normative data selected were similar with this cohort, the data were not age-matched. This approach has been previously documented in cohorts with *CRB1* [17]. A normative reference for BCEA 95% was set as 2.40 deg2 (SD ± 2.04, Range 0.2–11.7) [21]. In cases where the average reaction time was not available from the S-MAIA, the mean of the cohort was used. Statistical analysis comparing means between phenotypes was deemed unsuitable as small group sample sizes can lead to unreliable estimates of population parameters and increase the risk of Type I and Type II errors.

## 3. Results

### 3.1. Data Screening and Participant Compliance

**Figure 1:**
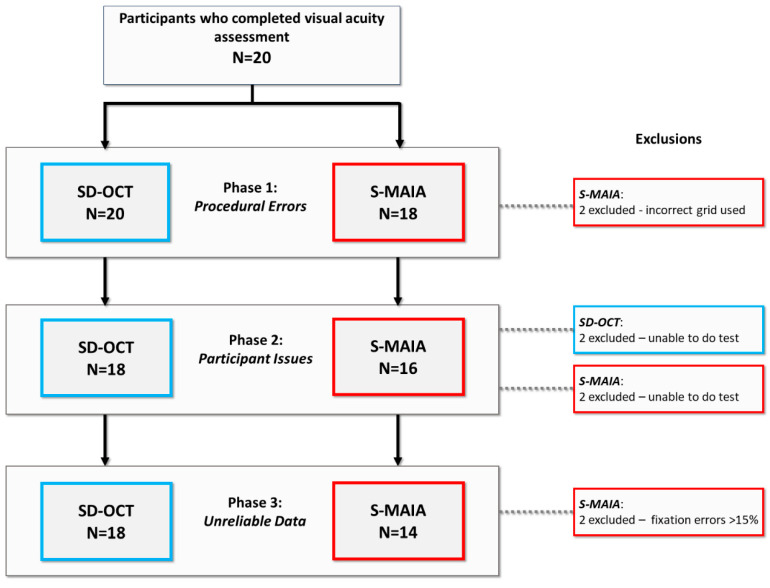
Flow diagram of data screening procedure and associated exclusions for SD-OCT and S-MAIA tests. Note: the same two participants were removed during phase 2 screening and were unable to undergo either SD-OCT or S-MAIA exam.

All participants recruited were able to have their BCVA assessed and recorded, meaning data from all 20 individuals with *CRB1*-associated retinopathies went through the screening process. Results from the data screening exercise for the participants are shown in the flow-chart in Figure 1. Two patients (10%) were excluded in screening phase 1 (procedural error) for the S-MAIA because the wrong grid was selected. Two participants (10%) were unable to complete both the SD-OCT and S-MAIA examinations and were excluded in screening phase 2 (participant issues). Lastly, two participants (10%) were unable to provide reliable data for the S-MAIA exam (fixation errors > 15%), and their data were excluded from data analysis. In summary, we have data for 18 participants in total; 18 participants with data from their SD-OCT assessment and 14 participants with data from their microperimetry assessments.

### 3.2. Demographic and Clinical Characteristics

Eighteen individuals with *CRB1*-associated retinopathies were assessed (88% white, 55% male), including 10 patients with MD, five with EOSRD/LCA and three with CORD. The age of onset for these conditions varied, ranging from 0.6 to 40 years. The median age of onset was 20 years, 4 years, and 10 years for MD, EOSRD/LCA, and CORD, respectively. When examining visual function of the study eye, the cohort displayed a median interquartile range (IQR) BCVA of 0.41 (0.32, 0.62) logMAR. The median BCVA (IQR) was 0.32 (0.32, 0.61) logMAR for MD, 0.46 (0.32, 0.5) logMAR for EOSRD/LCA, and 0.88 (0.61, 1.14) logMAR for CORD. For full demographic and clinical characteristics, see Table 1.

### 3.3. S-MAIA Analyses

Data were available for 14 participants for analysis: 8 participants with MD, 2 with CORD and 4 with EOSRD/LCA. For full details of S-MAIA parameters, see Table 2. Median (IQR) fixation losses for the whole cohort were 0 (IQR: 0.0). The median 95% bivariate contour ellipsoid areas (BCEA) score for the whole cohort was 2.15 deg^2^ (IQR: 1.43, 5.45), which is not significantly different to the normative reference (2.40 deg^2^, *p* = 0.9) [21]. The median rate of FP for the whole cohort was 0% (IQR: 0.0). Of the three participants who did exhibit wrong pressure events (a response occurring ≥1500 ms after a stimulus presentation and prior to the next stimulus presentation [14]), the median rate of FP was 9.06% (IQR: 8.53, 18.04). Correlational analysis was applied to the parameters from the microperimetry test and greater fixation losses were statistically significantly correlated with worse BCVA (*p* = 0.02; rho = −0.62) but not correlated with age. There were no other statistically significant correlations evidenced from the data.

All 14 participants were classed as having overall abnormal sensitivity, with no participants assessed as being suspect or normal. The median numbers for abnormal, suspect, and normal loci (out of 37 tested) were 34 (IQR: 29.25,37), 1 (IQR: 0,5.25), and 2 (IQR: 0,2.75), respectively. The severity of the loci tested were not correlated with BCVA or age. For the average numbers of abnormal, suspect, and normal loci per grouped by phenotype, see Table 2.

### 3.4. Eccentric Fixation

**Figure 2:**
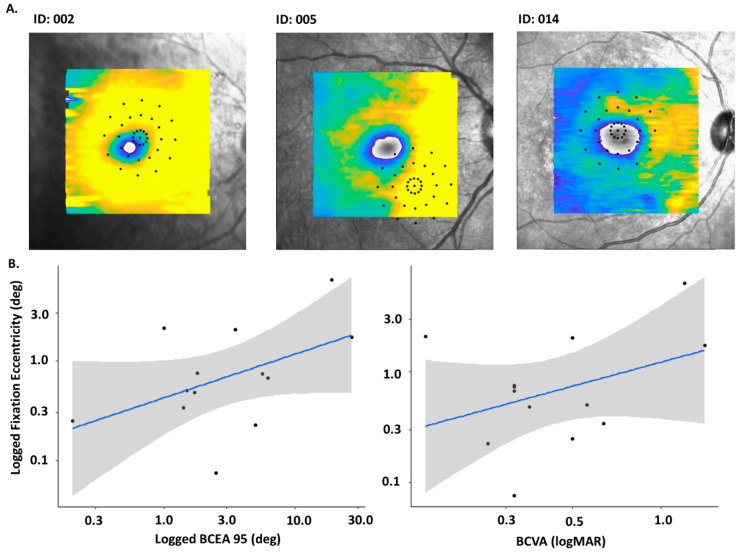
(**A**). Examples of eccentric fixation evident in the cohort when microperimetry grid data were overlayed on top of SD-OCT facing imaging. (**B**). Relation between eccentric fixation and BCEA 95% and BCVA in a cohort with *CRB1* Note: Participant 009 excluded due to epiretinal membrane presence.

The S-MAIA data were registered to the SD-OCT images using an in-house application and degrees of eccentric fixation were calculated (see Figure 2). As a proportion of data exhibited eccentric fixation, meaning between-group comparisons could not be made, structure-function analysis comparing SD-OCT data to S-MAIA data was not possible with these data. There were no discernible patterns of eccentricity within phenotypes or the cohort overall.

**Figure 3:**
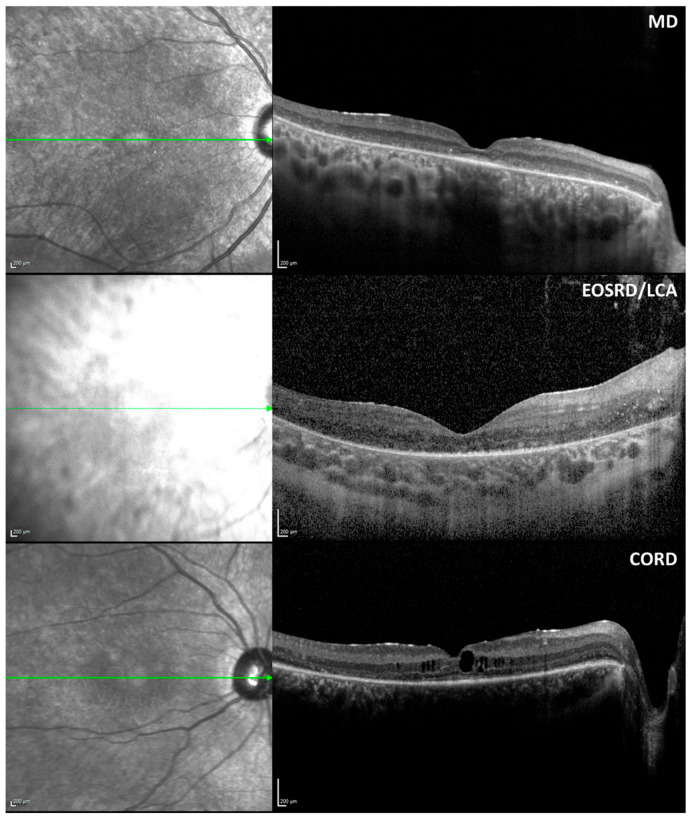
Examples of SD-OCT B-scans for each cohort. Participants 003, 002, and 007 are shown (from top to bottom).

Median (IQR) fixation eccentricity for the cohort was 0.70 deg (0.37,1.97). Linear regression was calculated to assess the relationships between eccentric fixation and BCEA 95% (R^2^ 0.23, *p* = 0.09), BCVA (R^2^ 0.25, *p* = 0.08), and foveal thickness (R^2^ 0.18, *p* = 0.15) but none were statistically significant (see Figure 3, note: Participant 009 was excluded from this analysis due to an epiretinal membrane, meaning the fovea could not be identified).

### 3.5. OCT Analyses

#### 3.5.1. Qualitative Analyses

In 18 participants with *CRB1*-associated retinopathies, the lamination of the retina was categorised into three grades. Four participants exhibited grade 1 (normal) lamination, nine participants had grade 2 lamination (normal organisation with coarse lamination), and five participants had grade 3 lamination (disorganisation with coarse lamination). Specifically, among participants with CORD, two had grade 2 and one had grade 3 lamination. Among those with EOSRD/LCA, one participant had grade 1 and four had grade 3 lamination. Participants with MD included three with grade 1 lamination and seven with grade 2 lamination. Additionally, cystoid macular oedema (CMO) was present in five participants: three with MD, one with CORD, and one with EOSRD/LCA. See Table 3 for OCT patterns across the different phenotypes and Figure 3 for examples.

#### 3.5.2. Foveal Thickness and Retinal Volume Analyses

**Figure 4:**
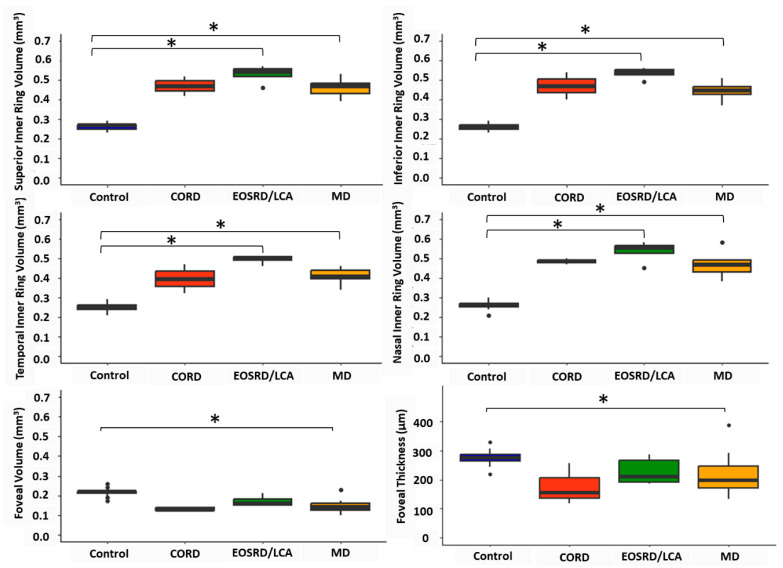
Retinal volume and foveal thickness from SD-OCT in participants and comparison to normative data [19,20]. Note: Statistically significant differences after correction for multiple comparisons are shown. * *p* < 0.05.

Participants with CMO were excluded from thickness assessment, leaving 13 participants. See Table 4 and Figure 4 for full details of foveal thickness and retinal volume assessment. Fovea thickness and volume (CRT1mm) were statistically significantly thinner than normative data (*p* < 0.01 and *p* < 0.001, respectively) [19,21].

The mean inner ring (*CRT1–3mm*) volume for the whole cohort was 0.47 mm^3^ (SD ± 0.06 mm^3^) and was significantly thicker (*p* < 0.001) when compared to the mean inner ring volume of the normative cohort (0.26 mm^3^ [SD ± 0.02 mm^3^]) [19]. The superior, inferior, nasal, and temporal quadrants had a statistically significantly larger volume when compared to normative data (all *p* < 0.001). There was no statistically significant relationship between mean inner ring volume and age, age of onset, or BCVA.

Following subgroup analysis, the mean inner ring volumes for MD, CORD, and EOSRD/LCA were 0.45 mm^3^ (SD ± 0.05 mm^3^), 0.46 mm^3^ (SD ± 0.07 mm^3^), and 0.52 mm^3^ (SD ± 0.04 mm^3^), respectively. For participants with MD, the superior, inferior, temporal, and nasal quadrants had a statistically significantly larger volume than normative data (all *p* < 0.001), while the foveal volume was significantly smaller (*p* < 0.05). For participants with EOSRD/LCA, the superior, inferior, temporal, and nasal quadrants had a statistically significantly larger volume than normative data (all *p* ≤ 0.01), while the foveal volume was not significantly smaller than the normative reference (*p =* 0.2). For the two participants with CORD, none of the quadrants nor the fovea were found to be significantly different from normative data.

## 4. Discussion

This study describes macular sensitivity using the S-MAIA and structure using SD-OCT data in individuals with genetically confirmed *CRB1*-associated retinopathies stratified into MD, EOSRD/LCA, and CORD phenotypes. Levels of participant attrition and associated reasons are reported. The analysis indicates that both macular sensitivity and retinal architecture in this cohort were abnormal compared to a visually healthy control population. Furthermore, SD-OCT data exhibited a significantly increased parafoveal volume, but a significantly reduced foveal volume and thickness compared to the normative reference. Yet, the presence of eccentric fixation posed a significant challenge, meaning a multimodal structure–function analysis was not possible.

Microperimetry serves as a widely adopted tool for evaluating treatment effectiveness in clinical trials related to various retinal dystrophies, including choroideremia [9] and RP [22]. Research supports the use of microperimetry for documenting and monitoring residual retinal function in *CRB1* patients [2,23]. This is the first time to the authors’ knowledge that the fast (screening) protocol on the S-MAIA has been used in patients with *CRB1*-associated retinopathies, which allows rapid assessment of sensitivity and is easily integrated into a clinical trial setting. The results revealed that all assessable participants with *CRB1*-associated retinopathies exhibited abnormal sensitivity. Specifically, an average of 34 out of 37 loci tested showed abnormal (<25 dB) sensitivity in the tested region. Yet, the levels of eccentric fixation in the cohort should be kept in mind. Fixation stability in the *CRB1* cohort was not significantly different from an established normal reference (2.40 deg^2^) [21], while a statistically significant correlation between greater number of fixation losses and worse BCVA was observed, emphasising the functional heterogeneity within the *CRB1* cohort. No other significant correlations were found between microperimetry parameters and BCVA or age. The relationship between increased fixation losses and deteriorating BCVA is unsurprising, given that disruptions in fixation often coincide with worsening BCVA due to the inability to maintain a steady gaze on a specific target [24] and people with more severe visual impairment often have more variable visual field outcomes [25].

In similar research, Nguyen et al. (2022) found a significant reduction in average sensitivity in a 2-year follow-up in 22 people with *CRB1*-associated retinopathies (86% with RP), while no change was identified in BCVA between visits [2]. While the number of fixation losses was not reported, BCEA 95% was reported to significantly correlate with BCVA, further illustrating unstable fixation is linked to visual acuity in people with *CRB1*-associated retinopathies. The authors also noted that sensitivity analysis was hindered by scotoma regions that expanded over time within the cohort, highlighting the inadequacy of relying solely on the average of all testing loci for accurate assessment in people with *CRB1*-associated retinopathies. While this study classified individual points as ‘normal’, ‘suspect’, or ‘abnormal’, no discernible patterns were observed. Hence, conducting pointwise sensitivity analysis across various luminance levels in a broad spectrum of *CRB1*-associated retinopathies could inform genotype–phenotype correlations and their changes over time, which are crucial for precise therapeutic interventions.

Comparatively, Roshandel et al. (2021) utilised both foveal and macular grid patterns and identified a wide range of microperimetry outcomes, with participants with *CRB1*-associated retinopathies exhibiting both disease progression and stable function over time. Particularly noteworthy was the observation in a young patient with RP of an increase in mean sensitivity, and the number of seeing loci was noted [23]. The heterogeneity of sensitivity loss patterns indicated by Roshandel et al. highlights more research is required in a larger cohort to identify the presence of potential *CRB1* phenotype-specific patterns. In addition, this variability observed in children highlights the need for age-specific considerations when conducting microperimetry assessments, and potentially alternative functional assessments should be explored alongside visual field evaluations. For example, Jones et al. (2016) assessed the feasibility of MAIA microperimetry in children and concluded while it was feasible, development of techniques to improve attentiveness and fixation is required [26].

The findings from this study were consistent with other literature regarding abnormal retinal architecture in *CRB1*-associated retinopathies [2,18]. Nguyen et al. reported 76% of patients had abnormal retina, including coarse lamination with or without disorganisation [2], and Varela et al. (2023) reported 65% of the assessed cohort had ill-defined lamination and 24% had disorganised retinal layers [17]. Similarly, abnormal retinal architecture was found in 78% of this cohort. A thickened retina has been associated with *CRB1* patients and reported previously in both retrospective studies using patient clinical data [17] and in animal models [27]. However, normal or thinned retinal thickness has also been previously described [28,29]. This study found that inner ETDRS ring retinal volume was significantly increased in the MD and EOSRD/LCA cohort compared to that of visually healthy controls, but this relation was not seen in the CORD groups (likely due to small sample size [n = 2]). While there is evidence for thickening in the perifoveal region [3,23], our findings indicate significant thickening in the inner ring (parafoveal region). No significant association was found between mean inner ring volume and age, age of onset, or BCVA. The authors speculate that this may be due to small sample size and the characteristic thickened, abnormal retina in these cohorts thinning over time, evidenced by longitudinal studies [23]. This lack of association between volume metrics and age has also been reported previously in EOSRD/LCA and MD cohorts [17].

Furthermore, this study reports a significantly reduced foveal volume and thickness in the MD cohort compared to the normative reference. While a similar trend in reduced foveal volume and thickness was evidenced in the EOSRD/LCA and CORD cohort, these were not significantly different to data from visually healthy controls. Roshandel et al. recently introduced the perifoveal-to-foveal volume ratio as a novel parameter, highlighting relative foveal thinning and perifoveal thickening even if both values are within normal ranges [23]. Our findings of a significant increase in volume in parafoveal region and reduced foveal volume support the utility of this ratio in capturing the structural changes in *CRB1*-associated retinopathies. These structural changes underscore the centralised degeneration observed in our cohorts, particularly in the MD group, and suggest that foveal thinning combined with perifoveal thickening might serve as important markers for disease progression and severity in *CRB1*-associated retinopathies.

In the context of clinical trials, assessing participant attrition is a critical aspect of study design and interpretation. High attrition rates can introduce bias and compromise the validity of study outcomes [30]. Moreover, understanding and addressing factors contributing to participant dropout, such as technical errors or participant-related issues, are essential for improving retention rates and enhancing the reliability of study findings. Therefore, implementing robust strategies to minimise participant attrition is paramount for the successful conduct of clinical trials focused on *CRB1*-associated retinopathies.

In this study, technical errors, participant-related issues, and unreliable data compromised a proportion of test results from the S-MAIA device (30%) and the SD-OCT (10%). These data were collected during a single visit, raising concerns that similar challenges may be compounded over repeat testing, particularly given the progressive nature of *CRB1*-associated retinopathies and other IRDs. In comparison, Nguyen et al. (2022) assessed macular sensitivity of 44 eyes with *CRB1*-associated retinopathies and reported 18% of the data unusable, while 16% were described as unreliable (due to age and/or severe visual impairment) and 2% were excluded following a technical error [2]. Retinal thickness was also assessed in 21 out of 22 eyes using SD-OCT and one eye was excluded due to limited participant co-operation, reportedly due to their young age (6 years) and nystagmus. This study identified a similar trend of issues contributing to unusable data and for similar reasons: participants unable to perform the task reliably or technical error. However, younger participants (aged 10–16 years) in this study performed better on the S-MAIA compared to older participants (aged 17–34) (see Table 1), contrasting with findings from Nguyen et al. Roshandel et al. (2021) examined both macular volume profile and microperimetry in *CRB1*-associated retinopathies in 10 participants, but 40% of data were excluded and associated reasons were not described. This highlights the severity of visual impairment in *CRB1* cohorts and the potential inability of some to conduct standardised clinical tests. These findings highlight the importance of carefully considering the FDA’s choice of microperimetry as a primary outcome measure [5,6], given its potential limitations in reliability and susceptibility to high attrition rates. Exploring alternative or complementary markers may enhance the ability to accurately assess disease progression in this population.

This study used a methodology which offers an accessible approach to gain a more accurate estimate of FP responses using data easily extracted from the device’s XML file, specifically for the fast (screening) protocol. This method was proposed by Montesano et al. (2021), who found the FP rate computed in this way exhibited a stronger correlation with test–retest variability compared to data inferred from indirect analysis of blind spot responses (BSR) [10]. It was found that FP rates were high in those who exhibited wrong pressure events, but this would be expected as the fast (screening) protocol is very short, naturally inflating the estimate. This methodology holds promise for translation into clinical practice due to its straightforward implementation, and researchers can readily apply the calculations to their own data extracted from S-MAIA, thereby enhancing the clinical utility of this approach.

Strengths of this study include its significant relevance as informing future *CRB1* clinical trials that aim to assess macular sensitivity and retinal thickness utilising the S-MAIA and SD-OCT. Additionally, a further strength of this study is that it highlights participant attrition using these devices in a clinical setting. By identifying and addressing factors contributing to participant dropout, such as technical errors or participant-related issues, researchers can improve retention rates and enhance the reliability of study outcomes. Furthermore, identifying the level of eccentric fixation and reporting reliability indices in this group is integral for those designing a clinical trial and plans to utilise the S-MAIA, not previously reported in similar studies in *CRB1* [2,23]. Moreover, this study assessed data from these devices only in participants who successfully had BCVA recorded, making these results representative of a study population that can adequately perform chart-based visual function assessment.

Despite its convenience and widespread clinical use, the S-MAIA fast (screening) testing protocol used in this study has limitations. Notably, it only evaluates field sensitivity at predetermined levels of 27 dB and 25 dB, categorising the area into ‘normal’, ‘suspect’, or ‘abnormal’. While it may not offer the same granularity as a full threshold test, which evaluates sensitivity thresholds across various luminance levels, the fast protocol still serves as a valuable tool for quickly identifying broad patterns of macular dysfunction. If the fast (screening) protocol of the S-MAIA is to be integrated into a clinical trial, it is essential to explore strategies to enhance a patient’s ability to consistently perform the test reliably and to ensure the testing grid is placed over the fovea. For instance, imaging data were used to manually locate the fovea for participants with *ABCA4*-associated retinal degeneration who had parafoveal fixation [31]. A similar procedure could be adopted for patients with *CRB1*-associated retinopathies and eccentric fixation. A further limitation of this study was that a practice test was not incorporated into the microperimetry testing protocol; thus, a learning affect may be evident in the data. Additionally, the small sample size in this study, though common in rare IRD research, limits the generalisability of the findings. Lastly, due to the eccentric fixation reported for this cohort, direct comparison between SD-OCT and S-MAIA data was not possible and assessment for regional sensitivity changes was unfeasible. By addressing these limitations, future research can enhance the accuracy and applicability of structure–function analysis in *CRB1*-associated retinopathies.

## 5. Conclusions

In conclusion, this study provides valuable insights into macular sensitivity and retinal architecture in individuals with *CRB1*-retinopathies, highlighting significant abnormalities compared to healthy controls. Specifically, foveal thinning combined with perifoveal thickening evidenced in the cohort support previous research on abnormal retinal architecture in *CRB1*-associated retinopathies. The findings also underscore the potential of combining S-MAIA microperimetry and SD-OCT imaging to assess disease severity and progression markers, despite challenges such as eccentric fixation and participant attrition. Addressing these methodological limitations in future research, such as by improved fixation training protocols and robust participant retention strategies, will be crucial in optimising clinical trial designs and improving outcomes for patients with *CRB1*-associated retinopathies.

## Figures and Tables

**Table 1:** Demographic characteristics of n = 18. Data sourced from self-reports.

ID	Gender	Ethnicity	Age (Years)	Family Number	Phenotype	Age of Onset (Years)	Zygosity	Variant 1 cDNA Variant 1 Protein	Variant 1 cDNA Variant 1 Protein	BCVA (logMAR)	Reliable Data from SD-OCT	Reliable Data from S-MAIA
001	F	Black	29	45590	MD	10	Homozygous	c.2506C>A p.Pro836Thr		0.32	Y	Y
002	F	White	17	46120	EOSRD/LCA	4	Compound Heterozygous	c.455G>A p.Cys152Tyr	c.3014A>T p.Asp1005Val	0.5	Y	Y
003	M	White	39	35083	MD	30	Compound Heterozygous	c.498_506del p.Ile167_Gly169del	c.4142C>G p.Pro1381Arg	0.64	Y	Y
004	F	White	48	43560	MD	30	Compound Heterozygous	c.1696G>T p.Glu556Ter	c.498_506del p.IIe167_Gly169del	0.16	Y	Y
005	M	White	47	38236	MD	24	Compound Heterozygous	c.498_506del p.Ile167_Gly169del	c.584G>T p.Cys195Phe	1.2	Y	Y
006	M	White	24	38229	EOSRD/LCA	6	Compound Heterozygous	c.2129A>T p.Glu710Val	c.3988del p.Glu1330Serfs*11	0.46	Y	N
007	F	Asian	13	Z88904	CORD	5	Compound Heterozygous	c.498_506del p.Ile167_Gly169del	c.4005+1G>A	0.36	Y	Y
008	M	White	15	-	MD	12	Compound Heterozygous	c.498_506del p.Ile167_Gly169del	c.1576C>T p.Arg525*	0.5	Y	Y
009	M	White	52	42270	MD	40	Compound Heterozygous	c.2401A>T p.Lys801*	c.498_506del p.Ile167_Gly169del	0.32	Y	Y
010	F	White	16	31953	EOSRD/LCA	<1	Compound Heterozygous	c.2548G>A p.Gly850Ser	c.4006-10A>G	0.32	Y	Y
011	M	White	17	37161	MD	8	Compound Heterozygous	c.498_506del p.Ile167_Gly169del	c.2308G>T p.Gly770Cys	0.02	Y	N
012	M	White	10	46830	EOSRD/LCA	2	Compound Heterozygous	c.2843G>A p.Cys948Tyr	c.1712A>C p.Glu571Ala	0.56	Y	Y
013	F	White	11	44092	MD	4	Compound Heterozygous	c.498_506del p.lle167_Gly169del	c.2843G>A p.Cys948Tyr	0.32	Y	Y
014	M	White	40	32038	CORD	20	Compound Heterozygous	c.498_506del p.IIe167_Gly169del	c.1431delG	1.4	Y	Y
015	M	White	29	33707	MD	18	Compound Heterozygous	c.498_506del p.IIe167_Gly169del	c.3827_3828del p.Glu1276Valfs*4	0.32	Y	Y
016	M	White	18	47941	EOSRD/LCA	5	Homozygous	c.2291G>A p.Arg764His		0.26	Y	Y
017	M	White	33	21819	CORD	6	Compound Heterozygous	c.470G>C p.C157Sp.Cys157Ser	c.2506C>A p.Pro836Thr	0.88	Y	N
018	F	White	34	35229	MD	25	Compound Heterozygous	c.2290C>T p.Arg764Cys	c.498_506del p.Ile167_Gly169del	0.86	Y	N

BCVA, best corrected visual acuity; EOSRD, early-onset severe retinal dystrophy; LCA, Leber congenital amaurosis; MD, macular dystrophy; CORD, cone-rod dystrophy; SD-OCT, spectral domain optical coherence tomography.

**Table 2:** Descriptive statistics of the parameters from the microperimetry test.

		MD (n = 8)	CORD (n = 2)	EOSRD/LCA (n = 4)	Whole Cohort (n = 14)
		Median (IQR)			
**Fixation Losses**		0 (0,0)	0 (0,0)	0 (0,3.25)	0 (0,0)
**BCEA 95% (deg^2^)**		1.6 (0.8,3.43)	14.35 (8.03,20.68)	4.25 (3,5.15)	2.15 (1.43,5.45)
Average Reaction Time * (ms)		660.79 (601.5,754.75)	699.39 (680.09,718.7)	715.39 (632.84,795.25)	660.79 (656.39,755)
**Duration (s)**		168 (161,173)	210.50 (193.25,227.75)	170 (163.75,188,75)	172.5 (163.25,176)
**Rate of FP (%)**		0 (0,2)	0 (0,0)	0 (0,2.3)	0 (0,0)
**Fixation Eccentricity (deg)**		0.71 (0.32,3.18)	1.10 (0.79,1.42)	0.62 (0.43,1.06)	0.70 (0.37,1.97)
**No.** **of** **Loci**	Abnormal (<25 dB)	33 (25.75,35.5)	36 (34.75,36.25)	34 (29.75,37)	34 (29.25,37)
	Suspect (≥25 dB, <27 dB)	3 (0.75,7.5)	1 (0.25,0.75)	1 (0,2.25)	1 (0,5.25)
	Normal (≥27 dB)	2 (0,3.25)	1 (0.5,1.5)	1 (0,3)	2 (0,2.75)

BCEA, bivariate contour ellipsoid areas; EOSRD/LCA, early-onset severe retinal dystrophy; FP, false positive; LCA, Leber congenital amaurosis; MD, macular dystrophy; CORD, cone-rod dystrophy; SD-OCT, spectral domain optical coherence tomography. * In the four cases where the average reaction time was not available from the S-MAIA, the mean of the cohort (660.79 ms) was used to calculate the rate of FP.

**Table 3:** SD-OCT patterns across the different phenotypes.

			MD (n = 10)	CORD (n = 3)	EOSRD/LCA (n = 5)	Whole Cohort (n = 18)
**Retina Lamination**		*Grade 1*	3	0	1	4
		*Grade 2*	7	2	0	9
		*Grade 3*	0	1	4	5
**CMO Presence**			3	1	1	5

EOSRD/LCA, early-onset severe retinal dystrophy; LCA, Leber congenital amaurosis; MD, macular dystrophy; CORD, cone-rod dystrophy; CMO, cystoid macular oedema.

**Table 4:** Retinal volume from OCT in participants and normative data.

	MD (n = 7)	CORD (n = 2)	EOSRD (n = 4)	Whole Cohort (n = 13)	Normative Reference
	Mean (SD)				
**Foveal Thickness (µm)**	191.71 (±51.44)	136.50 (±27.58)	214.25 (±36.65)	190.15 (±48.96)	275.16 (24.197)
**Foveal Volume (mm^3^)**	0.15 (±0.04)	0.13 (±0.01)	0.17 (±0.02)	0.15 (±0.04)	0.21 (±0.02)
**Superior Volume (mm^3^)**	0.46 (±0.05)	0.47 (±0.07)	0.53 (±0.05)	0.48 (±0.06)	0.26 (±0.02)
**Nasal Volume (mm^3^)**	0.47 (±0.07)	0.49 (±0.02)	0.54 (±0.06)	0.49 (±0.06)	0.26 (±0.02)
**Inferior Volume (mm^3^)**	0.44 (±0.05)	0.47 (±0.10)	0.54 (±0.03)	0.48 (±0.06)	0.26 (±0.01)
**Temporal Volume (mm^3^)**	0.41 (±0.04)	0.40 (±0.11)	0.50 (±0.02)	0.43 (±0.06)	0.25 (±0.02)

EOSRD, early-onset severe retinal dystrophy; LCA, Leber congenital amaurosis; MD, macular dystrophy; CORD, cone-rod dystrophy. Note: Data extracted from both the central portion and the 3 mm inner ring of the ETDRS grid.

## Data Availability

The summarised data presented in this study are provided in Table 1. Full datasets are available on request from the corresponding author.

## Appendix A

This method has been adapted from methodology proposed by Giovanni et al. (2021) but repurposed for the suprathreshold fast SMAIA programme [10]. The number of normal responses relates to how many times participants recorded seeing 27 dB, the number of suspect responses relates to how many times participants recorded seeing 25 dB, and number of abnormal responses relates to how many times participants recorded seeing or not seeing 27 dB or 25 dB.

### Appendix A.1. To Calculate Actual Test Time

((Number of normal responses + number of suspect responses) * ((average reaction time + 800 ms) + 150 ms)) + (number of suspect responses + (2 * number of abnormal responses)) * 1650 ms

### Appendix A.2. To Calculate Timecr

((Number of normal responses + number of suspect responses) * average reaction time) + (number of suspect responses + (2 * number of abnormal responses)) * 1500 ms

### Appendix A.3. To Calculate Timewr

Actual Test Time—Timecr

### Appendix A.4. To Calculate Rate of False Positives

λ = (Number WRs)/Timewr

FP = 1 − e − (λ * 1500 ms)
